# Testing Saliva to Reveal the Submerged Cases of the COVID-19 Iceberg

**DOI:** 10.3389/fmicb.2021.721635

**Published:** 2021-07-12

**Authors:** Elisa Borghi, Valentina Massa, Gianvincenzo Zuccotti, Anne L. Wyllie

**Affiliations:** ^1^Department of Health Sciences, Università degli Studi di Milano, Milan, Italy; ^2^Department of Biomedical and Clinical Sciences “L. Sacco”, Università degli Studi di Milano, Milan, Italy; ^3^Department of Epidemiology of Microbial Diseases, Yale School of Public Health, New Haven, CT, United States

**Keywords:** saliva, SARS-CoV-2, active surveillance, asymptomatic carriers, COVID-19

## Introduction

SARS-CoV-2 can spread from individuals before symptom onset and from asymptomatic individuals that are not aware of their infectious state. Compared with other deadly beta-coronaviruses, this occurs at a higher rate for SARS-CoV-2, allowing the virus to quietly diffuse through the community and to establish undetected reservoirs in the human population. While an estimated 7.5% of SARS-CoV cases and 9.8% of MERS-CoV (Wilder-Smith et al., 2005; Al-Tawfiq, 2020) cases remained asymptomatic, current estimates suggest that anywhere from 17 to 30% of SARS-CoV-2 positive subjects remain asymptomatic (Pollock and Lancaster, 2020; Johansson et al., 2021). As SARS-CoV-2 viral load in paucisymptomatic and asymptomatic subjects has been shown to not differ from those with symptoms (Yang et al., 2021), asymptomatic individuals can silently spread SARS-CoV-2 throughout their communities.

## Saliva For SARS-CoV-2 Infection Diagnosis

**Figure 1:**
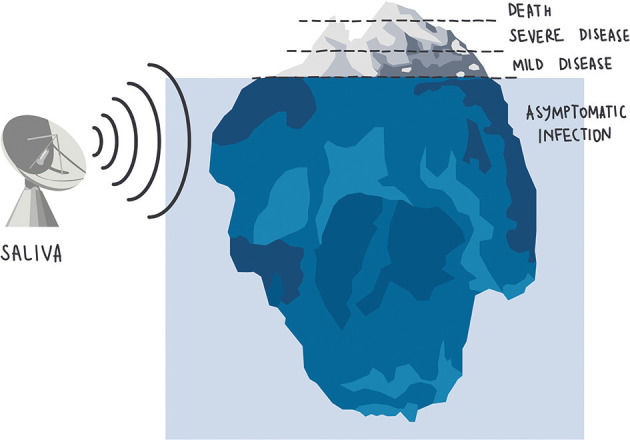
SARS-CoV-2 detection is sensitive and reliable in asymptomatic and paucisymptomatic subjects as well as in the early stages of the infection.

Thus, the “iceberg phenomenon,” highly recurrent in infectious diseases, has been dramatically demonstrated by SARS-CoV-2, and we appear to keep hitting the iceberg and sinking, despite the many ongoing efforts, worldwide. Literature suggests, however, that saliva could serve as an efficient radar allowing us to detect silent cases of SARS-CoV-2, helping to halt chains of transmission (Figure 1).

Compared to the gold standard nasopharyngeal swab, testing saliva for SARS-CoV-2 has been shown to have at least equal, and sometimes higher, sensitivity for detecting asymptomatic carriers (Savela et al., 2021; Yokota et al., 2021). Recent meta-analyses comparing the efficiency of PCR detection when applied to nasopharyngeal and saliva samples (Butler-Laporte et al., 2021; Cañete et al., 2021; Khiabani and Amirzade-Iranaq, 2021; Lee et al., 2021; Moreira et al., 2021), confirm the high specificity of saliva, with sensitivity positively correlating with stage of infection (i.e., early) and sampling technique (Tan et al., 2021).

Studies have demonstrated that SARS-CoV-2 RNA can appear in saliva 24–48 h prior to detection by nasopharyngeal swabs (Wyllie et al., 2020; Borghi et al., 2021), and 1.5–4.5 days prior to detection by anterior nasal swabs (Savela et al., 2021). In line with this, evidence also suggests that viral replication in the oral cavity may precede that within the nasopharynx, with multiple oral cells susceptible to SARS-CoV-2 infection (Huang et al., 2021). Indeed, its cellular receptor ACE2 (angiotensin-converting enzyme 2) has been detected in several oral epithelial cells including salivary glands (Matuck et al., 2021) and ducts, basal and suprabasal cells, mucous, and serous acini (Huang et al., 2021). These observations are not unique to SARS-CoV-2. Despite different primary target cells, saliva has also been reported as the first site of replication in the oral cavity for other highly infectious viruses, such as measles and Epstein-Barr (Jiang et al., 2006; Hutse et al., 2010). Additionally, a study in macaques on SARS-CoV, sharing the same entry receptor (ACE2), showed that viral loads in oral swabs was detectable in all animals at 48 h post-infection (Liu et al., 2011), suggesting a pivotal role of the oral cavity in early phase. Hence, saliva could represent the radar for the first warning sign useful both for halting the spreading and for patient management. It would be also important to verify its use for other biomarkers of SARS-CoV-2 infections, such as antibodies (Pisanic et al., 2020) or inflammatory mediators (Iebba et al., 2020).

## Saliva Collection and Processing Method Impacts the Test Sensitivity

Essential to an early warning system, however, are sensitive protocols of detection. Central to this are robust sampling methods, ensuring the collection of a high-quality sample for testing. As such, a major source of the variation reported for differences in test sensitivities, arises from differences in sample collection (Wyllie et al., 2020; Zou et al., 2020; Tan et al., 2021). Clear collection instructions are imperative; mucus contamination can make samples difficult to work with (Landry et al., 2020). True saliva thereby reduces biases from sample processing, increasing both the overall sensitivity of SARS-CoV-2 detection and the agreement with nasopharyngeal swab (Wyllie et al., 2020; Echavarria et al., 2021; Petrone et al., 2021). Many of the comparative studies however, show a reduced sensitivity when true saliva is self-collected by means of devices containing a transport or stabilizing media (Williams et al., 2020; Caulley et al., 2021; Dogan et al., 2021) likely at least in part a result from diluting the saliva sample. Since saliva has been demonstrated to self-preserve the detection of SARS-CoV-2 RNA (Ott et al., 2021), circumventing dilution increases the test sensitivity. By removing the need for additives, true saliva collection also permits the testing for SARS-CoV-2 directly in RT-qPCR, allowing labs to bypass non-organic nucleic acid extraction, thus streamlining the molecular analysis and test reporting (Borghi et al., 2021; Mahendra et al., 2021; Vogels et al., 2021).

While certain patient populations might experience a reduction in saliva production (da Silva Pedrosa et al., 2021), the use of devices to simultaneously stimulate and collect true saliva could represent a useful tool for enhancing sample collection. Any novel approach, however, must first be thoroughly tested on SARS-CoV-2 infected individuals, with virus RNA detection compared to that in a matched passive drool sample. Swab-based saliva collection, for example, may lead to inadequate saliva quantity while also potentially over-sampling limited regions of the mouth, reducing the chance of virus RNA detection (Manabe et al., 2021).

## Sampling Saliva Helps Overcoming Test Aversion to Swab-Based Approaches

The gold standard sample type for SARS-CoV-2 detection is still the nasopharyngeal swab. This method is considered an invasive sampling method, requiring specifically trained healthcare workers. The discomfort to those being sampled, not only poses a risk to healthcare workers should the patient sneeze or cough in response, but lowers testing compliance, decreasing its test sensitivity by default. As the pandemic progresses, the ability to self-collect saliva has the additional benefit of releasing health-workers for vaccines and non-COVID-19-related medical assistance.

## Conclusions

Despite vaccination programs around the world distracting from the continued need for SARS-CoV-2 screening, testing remains essential to protect the remaining susceptible individuals. Importantly, ongoing testing programs can provide insight into the risk for transmission in vaccinated individuals. Widespread implementation of saliva as a means for detecting the submerged portion of the SARS-CoV-2 iceberg should be considered essential for helping key decision makers avoid shipwrecks resulting from unseen virus circulation.

## Author Contributions

All authors listed have made a substantial, direct and intellectual contribution to the work, and approved it for publication.

## Conflict of Interest

The authors declare that the research was conducted in the absence of any commercial or financial relationships that could be construed as a potential conflict of interest.
